# Performance of critical care prognostic scoring systems in low and middle-income countries: a systematic review

**DOI:** 10.1186/s13054-017-1930-8

**Published:** 2018-01-26

**Authors:** Rashan Haniffa, Ilhaam Isaam, A. Pubudu De Silva, Arjen M. Dondorp, Nicolette F. De Keizer

**Affiliations:** 1 Centre for Tropical Medicine and Global Health, Nuffield Department of Medicine, University of Oxford, Oxford, UK; 2 Network for Improving Critical Care Systems and Training, Colombo, Sri Lanka; 3 AA (Ltd), London, UK; 4 National Intensive Care Surveillance, Ministry of Health, Amsterdam, Netherlands; 5 Mahidol–Oxford Tropical Medicine Research Unit, Faculty of Tropical Medicine, Mahidol University, Bangkok, Thailand; 6 Department of Medical Informatics, Academic Medical Center, Amsterdam, Netherlands

**Keywords:** Critical care, Prognostic model, ICU scoring system, Model performance, Low and middle-income countries, Resource-limited settings

## Abstract

**Background:**

Prognostic models—used in critical care medicine for mortality predictions, for benchmarking and for illness stratification in clinical trials—have been validated predominantly in high-income countries. These results may not be reproducible in low or middle-income countries (LMICs), not only because of different case-mix characteristics but also because of missing predictor variables. The study objective was to systematically review literature on the use of critical care prognostic models in LMICs and assess their ability to discriminate between survivors and non-survivors at hospital discharge of those admitted to intensive care units (ICUs), their calibration, their accuracy, and the manner in which missing values were handled.

**Methods:**

The PubMed database was searched in March 2017 to identify research articles reporting the use and performance of prognostic models in the evaluation of mortality in ICUs in LMICs. Studies carried out in ICUs in high-income countries or paediatric ICUs and studies that evaluated disease-specific scoring systems, were limited to a specific disease or single prognostic factor, were published only as abstracts, editorials, letters and systematic and narrative reviews or were not in English were excluded.

**Results:**

Of the 2233 studies retrieved, 473 were searched and 50 articles reporting 119 models were included. Five articles described the development and evaluation of new models, whereas 114 articles externally validated Acute Physiology and Chronic Health Evaluation, the Simplified Acute Physiology Score and Mortality Probability Models or versions thereof. Missing values were only described in 34% of studies; exclusion and or imputation by normal values were used. Discrimination, calibration and accuracy were reported in 94.0%, 72.4% and 25% respectively. Good discrimination and calibration were reported in 88.9% and 58.3% respectively. However, only 10 evaluations that reported excellent discrimination also reported good calibration. Generalisability of the findings was limited by variability of inclusion and exclusion criteria, unavailability of post-ICU outcomes and missing value handling.

**Conclusions:**

Robust interpretations regarding the applicability of prognostic models are currently hampered by poor adherence to reporting guidelines, especially when reporting missing value handling. Performance of mortality risk prediction models in LMIC ICUs is at best moderate, especially with limitations in calibration. This necessitates continued efforts to develop and validate LMIC models with readily available prognostic variables, perhaps aided by medical registries.

**Electronic supplementary material:**

The online version of this article (doi:10.1186/s13054-017-1930-8) contains supplementary material, which is available to authorized users.

## Background

Prognostic models used in critical care medicine for mortality predictions, for benchmarking and for illness stratification in clinical trials need to be validated for the relevant setting. An ideal model should have good discrimination (the ability to differentiate between high-risk and low-risk patients) and good calibration (generate risk estimates close to actual mortality) [1]. Acute Physiology and Chronic Health Evaluation (APACHE) or the Simplified Acute Physiology Score (SAPS) and the Mortality Probability Models (MPM) are some common prognostic systems used to predict the outcome of critically ill patients admitted to the intensive care unit (ICU) [2, 3].

The performance of these models has been extensively validated, predominantly in high-income countries (HICs) [4–6]. These results may not be reproducible in low or middle-income countries (LMICs), not only because of different case-mix characteristics but also because of missing predictor variables. Predictor variables that are routinely available in HIC ICUs (e.g. arterial oxygenation) are often not obtainable or reliable where resources are limited [7, 8]. Furthermore, data collection and recording may not be as robust in these settings as in HICs; paper-based recording systems, limited availability of staff and lack of staff training regarding data collection are frequent challenges [9]. The presence of missing values, if imputed as normal as per convention [3, 4, 10–13], will lead to underestimation of the scores and mortality. As part of quality improvement initiatives within ICUs, severity-adjusted mortality rates, which are calculated based on these prognostic systems, are increasingly used as tools for evaluating the impact of new therapies or organisational changes and for benchmarking; therefore, underestimating the risk could result in erroneous admission policies and an underestimation of the quality of care, performance and effectiveness when used for benchmarking [14]. Additionally, the diagnostic categories in these prognostic models may not be suited to capture diagnoses more common in these countries, such as dengue, malaria, snakebite and organophosphate poisoning. Furthermore, hospital discharge outcomes may not be readily accessible [15–17]. These and other factors influence the performance of the models, which may then require adjustment in the form of recalibration (adjustment of the intercept of the model and overall adjustment of the associations (relative weights) of the predictors with the outcome) and/or model revision (adjustment of individual predictor-outcome associations and addition or removal of new predictors) [18–20].

The objective of this article is to systematically review literature on the use of critical care prognostic models in LMICs and assess their ability to discriminate between survivors and non-survivors at hospital discharge of those admitted to ICUs, their calibration and accuracy, and the manner in which missing values are handled.

## Methods

### Literature search and eligibility criteria

The PubMed database was searched in March 2017, for research articles using the following search strategy: (*critical OR intensive*) *AND* (*mortality OR survival OR prognostic OR predictive*) *AND* (*scoring system OR rating system OR* APACHE *OR* SAPS *OR* MPM) in the title, abstract and keywords (Additional file 1).

No restrictions were placed on date of publication. Titles and abstracts returned were analysed for eligibility (RH, II). Abstracts reporting the performance of prognostic models were hand searched to identify studies carried out in ICUs in LMICs (as classified by the World Bank [21]) and full-text copies retrieved. Full-text articles were also retrieved when the title or abstract did not provide the country setting. The references of all selected reports were thereafter cross-checked for other potentially relevant articles.

The inclusion criteria for this review were studies carried out in ICUs in LMICs; those evaluating or developing prognostic models in adult ICU patients designed to predict mortality, whether ICU or hospital mortality.

The exclusion criteria for this review were: studies carried out only in ICUs in HICs or in paediatric ICUs; organ failure scoring systems such as SOFA that are not designed for predicting mortality; studies evaluating models in relation to a specific disease (e.g. liver cirrhosis) or limited to trauma patients; those assessing a single prognostic factor (e.g. microalbuminurea); studies published in languages other than English; studies published only as abstracts, editorials, letters and systematic or narrative reviews; and duplicate publications.

Where ICUs in both HICs and LMICs were included in a study, only data from the low/middle-income country were to be extracted. Likewise, where a single-factor or disease-specific scoring system and a non-specialty-specific scoring system were evaluated, only the data pertaining to the latter were extracted. Studies where both adult and paediatric patients were admitted to the same ICU and studies where the age limits of patients were not specified were to be included in this review.

### Data extraction and critical appraisal

The full-text articles were reviewed to assess eligibility for inclusion in the report. Disagreements between the two reviewers were resolved by discussion. The list of extracted items was based on the guidance issued by Cochrane for data extraction [22] and critical appraisal for systematic reviews of prediction models (the CHARMS checklist [23]). A second reviewer checked extracted items classed as “not reported” or “unclear”, or unexpected findings. If an article described multiple models, separate data extraction was carried out for each model.

### Descriptive analyses

Results were summarised using descriptive statistics. A formal meta-analysis was not planned as it was envisaged that the studies would be too heterogeneous, and a narrative synthesis was undertaken. Discrimination was assessed by the area under the receiver operating characteristic (AUROC) when reported [24]. Discrimination was considered excellent, very good, good, moderate or poor with AUROC values of 0.9–0.99, 0.8–0.89, 0.7–0.79, 0.6–0.69 and ≤ 0.6, respectively [25, 26]. Calibration was assessed by the Hosmer–Lemeshow *C* statistic (significant departures from perfect calibration were inferred when *p* values were less than 0.05 [24, 26]). Accuracy (the proportion of true positive and true negative in all evaluated cases [27]) was also considered.

## Results

### Study characteristics

**Fig. 1:**
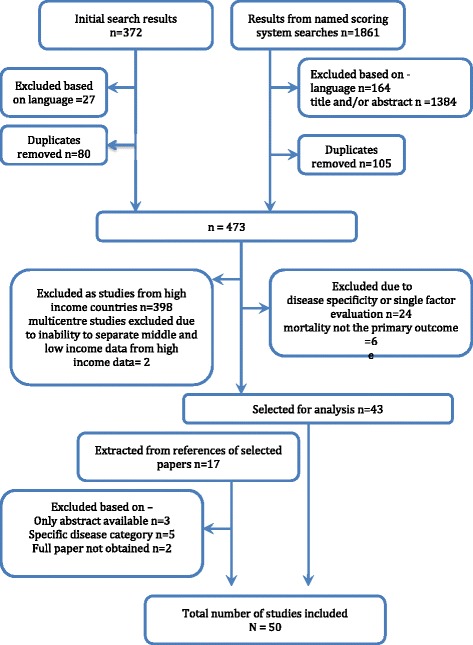
Study selection

Of the 2233 studies obtained from PubMed searches, 473 were searched and 43 met the inclusion criteria. Seven further studies were included after cross-checking the reference lists of the selected studies (Fig. 1). Fifty studies met the review criteria and were selected for analysis.

### Quality assessment

Study quality was assessed in accordance with the CHARMS guidelines [23] and is presented as Additional file 2. Variations existed in the conduct and reporting of the studies, especially with regard to inclusion and exclusion criteria, missing value handling, and performance and outcome measures.

Forty-three of the studies were carried out prospectively. The studies were carried out in 19 different LMICs, with the largest number carried out in India (studies = 11, models evaluated = 22), Thailand (studies = 6, models evaluated = 17) and Brazil (studies = 6, models evaluated = 17) (Table 1). Model adjustment was most frequent in India (*n* = 4 models). Settings, hospital and ICU characteristics are presented in Additional file 2.

**Table 1:** Study description

Study (country)	Scoring systems	Sample size (age in years)	(Outcome) and model performance measures	Study (country)	Scoring system	Sample size (age in years)	(Outcome) and model performance measures
Abhinandan and Vedavathi, 2013 (India) [60]	APACHE II	50 (18–90, mean 48.36)	(Unspecified mortality) D: not reported; C: not reported; CM: not reported; comparison of means	Liu et al., 2016 (China) [61]	APACHE II	137 (mean 69.53 ± 9.28)	(Hospital mortality) D: AUROC; C: not reported; CM: sensitivity, specificity
Aggarwal et al., 2006 (India) [7]	APACHE II SAPS II MPM II 0 MPM II 24	459 (16–80)	(Hospital mortality) D: AUROC; C: HL *C* and *H* statistics; CM: sensitivity, specificity, NPV, PPV, accuracy	Mohan et al., 2015 (India) [46]	APACHE II SAPS II SAPS 3	100 (mean: survivors 44.29 ± 15.53, non-survivors 57.37 ± 20.42)	(ICU mortality) D: not reported; C: not reported; CM: sensitivity, specificity; comparison of means
Ahluwalia et al., 1999 (India) [29]	APACHE II 48 hours (TA) New Score^a^	79 (13–80, mean 46)	(Hospital mortality) D: AUROC (CI not reported); C: not reported; CM: sensitivity, specificity	Nair et al., 2016 (India) [62]	SAPS	48 (mean: survivors 53.56, non-survivors 47.78)	(ICU mortality) D: AUROC; C: not reported
Celik et al., 2014 (Turkey) [63]	SAPS II	545 (>18)	(ICU mortality) D: not reported; C: not reported; CM: not reported; comparison of means	Naqvi et al., 2016 (Pakistan) [33]	APACHE II SAPS	96 (mean 32.93 ± 16.61)	(ICU mortality) D: AUROC; C: HL
Chang et al., 2006 (Taiwan) [64]	APACHE II	1263 (13–92, mean 56)	(Hospital mortality) D: AUROC; C: HL	Nassar et al., 2012 (Brazil) [42]	APACHE IV SAPS 3 MPM III	5780 (median 66, IQR 47–79)	(Hospital mortality) D: AUROC; C: HL *C* statistic
Chiavone and Rasslan, 2005 (Brazil) [65]	APACHE II	94 (16–97)	(Hospital mortality) D: AUROC; C: calibration curve, stratified in 10% risk bands, *R*^2^	Naved et al., 2011 (Pakistan) [34]	APACHE II (48 hours)	253 (15–84, mean 51.26 ± 17.9)	(ICU mortality) D: not reported; C: chi-square test
Eapen et al., 1997 (India) [30]	APACHE II (VA)	500 (13–84)	(Unspecified mortality) D: AUROC (CI not reported); C: not reported	Nimgaonkar et al., 2004 (India) [35]	APACHE II ANN22^a^ ANN15^a^	2962 (mean 37.5 ± 16.1)	(Hospital mortality) D: AUROC; C: HL
Evran et al., 2016 (Turkey) [47]	APACHE II APACHE IV SAPS 3	487 (18–96, mean 58.58 ± 18	(ICU mortality) D: AUROC (as figure); C: not reported; CM: sensitivity, specificity, accuracy, PPV and NPV	Nouira et al., 1998 (Tunisia) [66]	APACHE II SAPS II MPM 0 MPM 24	1325 (mean 46.6 ± 20.4)	(Hospital mortality) D: AUROC; C: HL *C* and *H* statistics; CM: NPV, PPV
Fadaizadeh et al., 2012 (Iran) [67]	APACHE II SAPS II	415 (mean 49.28 ± 0.94)	(ICU mortality) D: AUROC; C: HL *C* statistic; CM: sensitivity, specificity	Ratanarat et al., 2005 (Thailand) [68]	APACHE II SAPS I	482 (mean 56 ± 21)	(ICU mortality) D: AUROC; C: not reported CM: sensitivity, specificity
Faruq et al., 2013 (Bangladesh) [37]	APACHE II SAPS II	194 (mean 61.06 ± 15.42	(ICU mortality) D: AUROC; C: HL *C* statistic	Riviello et al., 2016 (Rwanda) [43]	MPM III (0) R-MPM^a^ Simplified R-MPM^a^	427 (median 34, IQR 25–47)	(Hospital mortality) D: AUROC; C: HL
Galal et al., 2013 (Egypt) [49]	APACHE II SAPS II	105 (0–88, mean 54.59 ± 15.75)	(ICU mortality) D: AUROC (CI not reported); C: HL; CM: sensitivity, specificity, accuracy	Sathe and Bapat, 2014 (India) [69]	APACHE II SAPS II	1543 (mean 53.9 ± 16.8)	(Hospital mortality) D: AUROC; C: HL
Gilani et al., 2014 (Iran) [70]	APACHE II APACHE III SAPS II	202 (14–85 mean 53.1 ± 20.3)	(Hospital mortality) D: AUROC (CI not reported); C: HL; CM: sensitivity, specificity, accuracy, NPV, PPV	Sekulic et al., 2015 (Serbia) [48]	APACHE II MPM II (0) MPM II (24) MPM II (48) MPM II (72) MPM II (7 days) SAPS II	60 (mean 59.0 ± 15.8)	(ICU mortality) D: AUROC (as figure for MPM II (24)); C: HL; CM: sensitivity, specificity
Godinjak et al., 2016 (Bosnia and Herzegovina) [31]	APACHE II SAPS II	174 (19–87, mean 61.7 ± 16.3)	(Hospital mortality) D: AUROC; C: not reported; CM: sensitivity, specificity	Shoukat et al., 2016 (Pakistan) [71]	APACHE IV	155 (13–70, mean 38.39 ± 13.61)	(Unspecified mortality) D: not reported; C: not reported; CM: not reported; comparison of means
Gupta and Arora, 2004 (India) [72]	APACHE II	330 (mean 43.32 ± 16.22)	(ICU mortality) D: AUROC (CI not reported); C: HL; CM: accuracy	Shrestha et al., 2011 (Nepal) [73]	APACHE III	117 (16–84, mean 43.18 ± 18.49)	(ICU mortality) D: AUROC; C: HL; CM: sensitivity, specificity, NPV, PPV, accuracy
Haidri et al., 2011 (Pakistan) [74]	APACHE II	142 (15–90, mean 53.16 ± 19.29)	(ICU mortality) D: not reported; C: not reported; comparison of means	Silva Junior et al., 2010 (Brazil) [55]	SAPS 3	1310 (mean 67.1 ± 15.3)	(Hospital mortality) D: AUROC; C: HL *C* statistic; CM: sensitivity, specificity
Halim et al., 2009 (Indonesia) [75]	APACHE II	144 (15–85, mean 47.33 ± 17.17)	(ICU mortality) D: AUROC (CI not reported); C: HL; CM: sensitivity, specificity, accuracy, NPV, PPV	Soares and Salluh, 2006 (Brazil) [39]	SAPS II SAPS 3 SAPS 3 (CSA)	952 (>18, mean 58.3 ± 23.1)	(Hospital mortality) D: AUROC; C: HL *C* statistic
Hamza et al., 2009 (Egypt) [76]	APACHE IV SAPS II MPM II0	265 (mean 57.07 ± 16.6)	(ICU mortality) D: AUROC; C: HL *H* statistic	Soares et al., 2004 (Brazil) [40]	APACHE II APACHE III SAPS II, MPM II 0 MPM II 24	1257 (18–93, mean 56.0 ± 16.7)	(Hospital mortality) D: AUROC; C: HL *H* statistic
Hashmi et al., 2016 (Pakistan) [77]	APACHE II APACHE II (MTA)	213 (mean 46.31 ± 18.43)	(Unspecified mortality) D: AUROC; C: HL; CM: sensitivity, specificity, accuracy	Soares et al., 2010 (Brazil) [41]	SAPS II SAPS 3 SAPS 3 (CSA) MPM III (24)	717 (mean 61.2 ± 15.4)	(Hospital mortality) D: AUROC; C: HL *C* statistic
Hernandez et al., 2014 (Philippines) [78]	SAPS 3	2426 (mean 62 ± 17)	(ICU mortality) D: AUROC (CI not reported); C: HL *C* statistic	Sutheechet, 2009 (Thailand) [79]	SAPS II MPM II (24)	639 (18–100, mean 56.9 ± 20.6)	(Hospital mortality) D: AUROC; C: HL; CM: sensitivity, specificity, accuracy, NPV, PPV
Hosseini and Ramazani, 2015 (Iran) [80]	APACHE II	150 (3–97)	(ICU mortality) D: AUROC; C: HL; CM: sensitivity, specificity, accuracy	Teoh et al., 1991 (Malaysia) [81]	APACHE II	100 (1 month–82 years)	(Hospital mortality) D: not reported; C: not reported; CM: not reported; correlation
Juneja et al, 2012 (India) [1]	APACHE II APACHE III APACHE IV SAPS II SAPS 3 MPM II (0) MPM III (0)	653 (mean 58.48 ± 18.6)	(ICU mortality) D: AUROC; C: HL; CM: sensitivity, specificity	Turner et al., 1989 (South Africa) [36]	APACHE II	728 (12–88, mean 43)	(Unspecified mortality) D: not reported; C: not reported; CM: not reported; correlation
Khan et al., 2015 (India) [32]	APACHE II APACHE II (48 hours) SAPS II SAPS II (48 hours)	85 (mean 39.14 ± 17.3)	(Hospital mortality) D: AUROC; C: not reported; CM: sensitivity, specificity	Wilairatana et al., 1995 (Thailand) [82]	APACHE II APACHE III SAPS II	209 (16–93, mean 55.36 ± 17.44)	(Hospital mortality) D: AUROC; C: not reported; CM: sensitivity, specificity, accuracy
Khawannimit and Geater, 2007 (Thailand) [38]	APACHE II SAPS II	1316 (mean 55.6 ± 18.2)	(Hospital mortality) D: AUROC; C: HL *C* and *H* statistics; CM: sensitivity, specificity, NPV, PPV, accuracy	Xing et al., 2015 (China) [52]	APACHE II APACHE IV SAPS 3	981 (mean 64.8 ± 12.1)	(Hospital mortality) D: AUROC; C: HL *C* statistic
Khwannimit and Bhurayanontachai, 2011 (Thailand) [51]	APACHE II SAPS 3 SAPS 3 (AUS) SAPS II Customised APACHE II (recalibration) Customised SAPS II (recalibration) Customised SAPS 3 (recalibration)	2022 (median 62, IQR 49–73)	(Hospital mortality) D: AUROC; C: HL *H* and *C* statistic	Yamin et al., 2011 (Pakistan) [28]	APACHE IV	162 (9–90, mean 38.024)	(ICU mortality) D: not reported; C: not reported; CM: accuracy; comparison of means
Kiatboonsri and Charoenpan, 1995 (Thailand) [45]	APACHE II	334 (15–98, mean 56.01 ± 18.23)	(Hospital mortality) D: AUROC not reported; C: not reported; CM: sensitivity, specificity, PPV, accuracy	Zhao et al., 2013 (China) [50]	SAPS II (initial) SAPS II (48 hours) SAPS II (24 hours) SAPS II (72 hours) Simplified SAPS II (VA)	1684 (18–98, mean 58.93 ± 18.30)	(ICU mortality) D: AUROC; C: HL; CM: sensitivity, specificity, accuracy; correlation

^a^ New model development

*VA* variable adjustment, *MTA* modelling technique adjustment, *D* discrimination, *AUROC* area under the receiver operating characteristic curve, *C* calibration, *HL* Hosmer–Lemeshow statistic, *CM* classification measures (e.g. sensitivity, specificity, *PPV* positive predictive values, *NPV* negative predictive values, *APACHE* Acute Physiology and Chronic Health Evaluation, *ANN* artificial neural network, *SAPS* Simplified Acute Physiology Score, *SAPS (AUS)* Simplified Acute Physiology Score (Australian), *SAPS (CSA)* Simplified Acute Physiology Score (Central and South American), *MPM* Mortality Probability Models, *R-MPM* Rwanda-Mortality Probability Model, *ICU* intensive care unit, *IQR* interquartile range, *CI* confidence interval

Sample sizes ranged from 48 to 5780, and participant ages ranged from 1 month to 100 years (Table 1). Of the 33 studies reporting a lower age limit, 17 reported participants under the age of 18 years (Table 1).

Missing value handling was explicitly mentioned in 17 studies (Table 2). One study reported incomplete data for 26.4% of its patients but did not provide details on how this was handled [28]. Patients were excluded in nine of the studies [28–36], normal physiological values were imputed in five studies [37–41] and both exclusion (for missing variables such as chronic health status) and imputation by normal (for missing physiological values) occurred in two studies [42, 43]. No other methods of imputation were described. For the most commonly assessed models (APACHE II, SAPS II and SAPS 3) missing values were mentioned only 34.1%, 31.0% and 42.9% of the time respectively.

**Table 2:** Missing value handling

Study	Scoring system/s	Missing value handling
Exclusion		
Celik et al. (2014) [63]	SAPS II	178 (21.1%) were excluded due to lack of data, and 46 (5.55%) patients were excluded due to archival documentation problems. No information on admission source, attached devices, PaO_2_/FiO_2_ and Glasgow Coma Score was available for the excluded group in the computerised medical records. The included group survey did not differ from the excluded group regarding age, gender, admission time and admission day. The prevalence of trauma and intoxication was higher among the excluded group than the included group (trauma 15%, intoxication 30%)
Chiavone et al. (2005) [65]	APACHE II	One patient excluded
Godinjak et al. (2016) [31]	APACHE II SAPS II	15 patients (7.9%) who died in the first 24 hours after admission to the MICU
Haidri et al. (2011) [74]	APACHE II	All patients with incomplete records and missing variables including laboratory investigations or who were not followed up due to any reason were excluded
Hernandez et al. (2014) [78]	SAPS 3	159 (6.6%) were excluded for incomplete SAPS 3 data
Naqvi et al. (2016) [33]	APACHE II SAPS	29 patients (23.6%) with incomplete information of scoring system in case records
Naved et al. (2011) [34]	APACHE II	Patients with incomplete records not included (numbers not reported)
Willairatna et al. (1995) [82]	APACHE II APACHE III SAPS II	When scores could not be derived due to an incomplete set of physiological data, patients were excluded
Normal value imputation		
Faruq et al. (2013) [37]	APACHE II SAPS II	GCS attributed as normal
Khwannimit and Geater (2007) [38]	APACHE II SAPS II	GCS attributed as normal Missing physiological variables were found in only 6% for APACHE II (excluding bilirubin, which was missing in 76.5% of the presented data records) and 6.3% for SAPS II variables (excluding bilirubin, which was missing in 76.5% of the presented data records)
Soares et al. (2004) [40]	APACHE II APACHE III MPM II (0) MPM II (24) SAPS II	Zero points or normal values were inserted where data were missing [19]. There were no missing variables for physiological data. Among laboratory variables, normal values were inserted for albumin in 623 (49.6%), prothrombin time in 274 (21.8%) and bilirubin in 676 (53.8%) patients. No patient with jaundice on physical examination lacked serum bilirubin measurements
Soares and Salluh (2006) [39]	SAPS II SAPS 3 SAPS 3 (CSA)	Zero points or normal values were assigned for missing variables [1, 12]. There were no missing data for demographic, clinical and physiologic data. Among laboratory variables, normal values were attributed only for bilirubin in 535 patients (56%). No patient with jaundice lacked bilirubin level measurements
Soares et al. (2010) [41]	MPM III (24) SAPS II SAPS 3 SAPS 3 (CSA)	Zero points or normal values were attributed for missing variables. There were no missing data for demographic, clinical and physiologic data. Missing laboratory variables are depicted in Table 1 of Electronic Supplementary Material [41]. No patient with jaundice lacked bilirubin level measurements
Exclusion and normal value imputation		
Nassar et al. (2012) [42]	APACHE IV SAPS 3 MPM III	3.02% patients with incomplete data which prevented adequate calculation of one or more of the scores were excluded; these missing data could be pre-ICU length of stay, reason for ICU admission, chronic health variables and mechanical ventilation on first day. Missing physiologic variables, namely bilirubin, acid-base abnormalities, PaO_2_ or PaO_2_/FiO_2_ ratio, were considered as normal for purpose of calculations

*APACHE* Acute Physiology and Chronic Health Evaluation, *SAPS* Simplified Acute Physiology Score, *SAPS (CSA)*, Simplified Acute Physiology Score (Central and South American), *MPM* Mortality Probability Models, *GCS* Glasgow Coma Score, *ICU* intensive care unit, *MICU* medical intensive care unit, *PaO*_*2*_ partial pressure arterial oxygen, *FiO*_*2*_ fraction of inspired oxygen

### Model performance

The 50 studies reported a total of 114 model performance evaluations for nine versions of APACHE, SAPS and MPM as described in the subsection ‘Evaluation of the performance of existing models’. Three of the analysed studies [29, 35, 43] also described the development of five new prediction models in LMIC settings. These five new models are presented separately.

#### Evaluation of the performance of existing models

Model performance is described in the following in terms of the performance of the individual model evaluations carried out (*n* = 114).

External evaluation of models (model performance evaluation on a related but different population than the population on which the model has originally been developed [44]) was carried out 108 times as follows: performance of APACHE II was evaluated 36 times, of APACHE III five times, of APACHE IV seven times, of SAPS I twice, of SAPS II 26 times, of SAPS 3 13 times, of MPM I twice, of MPM II 12 times and of MPM III five times (Table 1).

Model adjustment was carried out six times (Table 3): three models were recalibrated using first-level customisation (computing a new logistic coefficient, while maintaining the same variables with the same weights as in the original model); two models were revised by the exclusion and/or substitution of variables; and one evaluation altered the way in which APACHE II was calculated—from the usual manual method to automatic calculation using custom-built software.

**Table 3:** Model adjustment and performance

Study	Type of adjustment and changes made	Discrimination (original scoring system)	Discrimination(after adjustment)	Calibration (original scoring system)	Calibration (after adjustment)
APACHE II					
Khwannimit and Bhurayanontachai (2011) [51]	Recalibration (first-level customisation): customised APACHE II logit = –7.7206 + (APACHE II score × 0.2013) + new diagnostic category weight (Appendix I [51])	0.936 (0.925–0.947) (entire population *n* = 2022)	0.936 (0.925–0.947) (validation dataset *n* = 1011)	*C* statistic χ^2^ = 104.2 (*p* = 0.001), *H* statistic χ^2^ = 113.1 (*p* < 0.001)	*C* statistic χ^2^ = 16.1 (*p* = 0.09), *H* statistic χ^2^ = 14.1 (*p* = 0.17)
Eapen et al. (1997) [30]	Variable adjustment: GCS excluded	Not evaluated	0.6068	Not reported	Not reported
Hashmi et al. (2016) [77]	Modelling technique adjustments: APACHE II calculated automatically by software which uses manually entered values using the logit equation = –4.063 + (APACHE II) × 0.181	0.823 (0.76–0.88) (manual calculation)	0.827 (0.77–0.88) (software calculation)	χ^2^ = 11.76 (*p* = 0.16)	χ^2^ = 5.46 (*p* = 0.71)
Nimgaonkar et al. (2004) [35]	Modelling technique adjustments: Artificial Neural Network (ANN 22) model trained on an Indian patient dataset using all 22 APACHE II variables	0.77	0.87 (*p* < 0.002)	*H* statistic χ^2^ = 123.5 (*p* < 0.05)	*H* statistic χ^2^ = 22.4 (*p* < 0.05)
Nimgaonkar et al. (2004) [35]	Modelling technique adjustments: Artificial Neural Network (ANN 15) model trained on an Indian patient dataset using 15 APACHE II variables	0.77	0.88 (*p* < 0.001) ANN 15	*H* statistic χ^2^ = 123.5 (*p* < 0.05)	*H* statistic χ^2^ = 27.7 (*p* < 0.05)
SAPS II					
Khwannimit and Bhurayanontachai (2011) [51]	Recalibration (first-level customisation): customised SAPS II logit = –10.1779 + 0.0719 (SAPS II score) + 1.4891 × ln(SAPS II score + 1)	0.914 (0.901–0.928) (entire population *n* = 2022)	0.919 (0.900–0.938) (validation dataset *n* = 1011)	*C* statistic χ^2^ = 124.9 (*p* < 0.001), *H* statistic χ^2^ = 97.5 (*p* < 0.001)	*C* statistic χ^2^ = 8.6 (*p* = 0.57), *H* statistic χ^2^ = 9.6 (*p* = 0.48)
Zhao et al. (2013) [50]	Variable adjustment: 1. Underlying disease variables excluded 2. Admission type variables excluded	0.776 (95% CI 0.750–0.802) at admission, 0.826 (95% CI 0.803–0.850) at 24 hours	Not reported: correlation was suggested between the simplified SAPS II score at each time point and outcome with OR of 1.109 (*p* = 0.000), regardless of the diagnosis	Not reported	Not reported
SAPS 3					
Khwannimit and Bhurayanontachai (2011) [51]	Recalibration (first-level customisation): customised SAPS 3 logit = –33.4249 + ln(SAPS 3 score +1) × 7.8699	0.913 (0.899–0.924) (entire population *n* = 2022)	0.917 (0.897–0.937) (validation dataset *n* = 1011)	*C* statistic χ^2^ = 170 (*p* < 0.001), *H* statistic χ^2^ = 79.9 (*p* < 0.001)	*C* statistic χ^2^ = 8.2 (*p* = 0.61), *H* statistic χ^2^ = 79.9 (*p* < 0.001)
Riviello et al. (2016) [43]	MPM (0) III	Exclusion of two patients (0.5%) due to lack of discharge vital status Normal values attribution details provided in Supplementary Table 3 of the original paper. Highest proportions of missing values were for GCS (36.30%) followed by chronic renal compromise/insufficiency (7.96%)			

*APACHE* Acute Physiology and Chronic Health Evaluation, *SAPS* Simplified Acute Physiology Score, *MPM* Mortality Probability Models, *GCS* Glasgow Coma Score, *ICU* intensive care unit, *CI* confidence interval, *OR* odds ratio

The mortality endpoint assessed for 60 (52.6%) of the performance evaluations was hospital or post-hospital mortality; for 47 (41.2%) evaluations it was ICU mortality and for seven (6.1%) the mortality endpoint was not specified (Table 1).

Ten (6%) model performance evaluations did not report either discrimination or calibration. The methods used for evaluation are presented in Table 4.

**Table 4:** Model performance where discrimination was not reported

Study	Model	Performance
Abhinandan and Vedavathi (2013) [60]	APACHE II	Student *t* test
		Although APACHE II score was higher among non-survivors than survivors (23.28 vs 18.75), it was just statistically significantly with *p* = 0.068+
Haidri et al. (2011) [74]	APACHE II	Comparison of means between those who survived and those who died
		The mean 24 h APACHE II score of those who were discharged was 18.93 ± 7.19 and that of those who died was 22.33 ± 7.80.
Mohan et al. (2015) [46]	APACHE II	30% of patients with APACHE II score < 14 died (unadjusted relative risk = 1.00) and 68.3% with score > 14 died (relative risk = 2.6 (95% CI 1.5–2.7), *p* < 0.001.
Naved et al. (2011) [34]	APACHE II (48 hours)	Chi-square test
		Significant relationship between outcome and APACHE II score (χ^2^ = 58.7, *p* = 0.001)
Teoh et al. (1991) [81]	APACHE II	APACHE II scores were correlated with hospital mortality (bar graph)
		Mortality was higher with a higher APACHE II score. There were no deaths in the 0–4 APACHE II score group. In higher ranges of APACHE II score of 30 onwards there was a 100% mortality, except for APACHE II score of 45–49 for which there were no admissions within this group
Turner et al. (1989) [36]	APACHE II	APACHE II scores were correlated with hospital mortality (bar graphs plotted)
Shoukat et al. (2016) [71]	APACHE IV	The mortality increased with an increase in APACHE IV score (scores vs mortality presented as bar graph). All patients with score more than 39 did not survive
Yamin et al. (2011) [28]	APACHE IV	Mean predicted mortality of overall patient was found to be 25.7% while observed mortality was 28.4% with SD of 0.439 and SMR = 1.09. 62.1% of the overall population show the same outcome as predicted by APACHE IV (*p* = 0.61)
Celik et al. (2014) [63]	SAPS II	Student *t* test
		Mean SAPS II score of the patients who died (59.37 ± 16.50) was significantly higher than that of the patients who were discharged (33.70 ± 13.90) (*t* = 18.85, *p*= 0.000).
Zhao et al. (2013) [50]	Simplified SAPS II	A correlation was suggested between the simplified SAPS II score at each time point and outcome with OR of 1.109 (*p* = 0.000), regardless of the diagnosis

*APACHE* Acute Physiology and Chronic Health Evaluation, *SAPS* Simplified Acute Physiology Score, *CI* confidence interval, *SD* standard deviation, *OR* odds ratio, *SMR* standardised mortality ratio

Tables 5, 6 and 7 describe the model performance of all versions of APACHE, SAPS and MPM respectively in terms of discrimination, calibration and accuracy.

**Table 5:** Model performance for all versions of APACHE

Study	Scoring system	Discrimination	Calibration	Sensitivity	Specificity	Accuracy
Khwannimit and Bhurayanontachai (2011) [51]	APACHE II (recalibrated model)	0.936 (0.925–0.947)	*C* statistic χ^2^ = 16.1 (*p* = 0.09)	NR	NR	NR
			*H* statistic χ^2^ = 14.1(*p* < 0.17)			
Khwannimit and Bhurayanontachai (2011) [51]	APACHE II	0.936 (0.925–0.947)	*C* statistic χ^2^ = 104.2(*p* < 0.001)	NR	NR	NR
			*H* statistic χ^2^ = 113.1(*p* < 0.001)			
Khan et al. (2015) [32]	APACHE II (48 hours)	0.933 (0.873–0.992)	NR	94.1% (DC > 9.5)	86.3% (DC > 9.5)	NR
Godinjak et al. (2016) [31]	APACHE II	0.920 (0.87–0.97)	NR	74.5% (DC = 27.5)	93.4% (DC = 27.5)	NR
Khawannimit and Geater (2007) [38]	APACHE II	0.911 (0.891–0.93)	*C* statistic χ^2^ = 66.65 (*p* < 0.001)	73.87% (95% CI = 65.23–75.66)	92% (95% CI = 89.66–93.20)	87% (95% CI = 85.47–89.13)
			*H* statistic χ^2^ = 66.59 (*p* < 0.001)			
Fadaizadeh et al. (2012) [67]	APACHE II	0.897 (0.858–0.937)	*C* statistic χ^2^ = 3:27 (*p* = 0:916)	90% (DC = 13.5)	75% (DC 13.5)	NR
Juneja et al. (2012) [1]	APACHE II	0.894 (0.864–0.925)	χ^2^ = 7.959 (*p* = 0.438)	74.8% (DC >20.5)	84.9% (DC >20.5)	NR
Soares et al. (2004) [40]	APACHE II	0.888 (0.868–0.907)	*H* statistic χ^2^ = 78.181 (*p* < 0.001)	NR	NR	NR
Xing et al. (2015) [52]	APACHE II	0.863 (0.804–0.923)	χ^2^ = 3.486 (*p* = 0.900)	NR	NR	NR
Sathe and Bapat (2014) [69]	APACHE II	0.86	χ^2^ = 12.8 (*p* = 0.03)	NR	NR	NR
Hosseini and Ramazani (2015) [80]	APACHE II	0.857 (0.788–0.925)	χ^2^ = 10.203 (*p* = 0.251)	96.6%	62.80%	79.70%
Naqvi et al. (2016) [33]	APACHE II	0.835	χ^2^ = 3.199 (*p* = 0.866)	NR	NR	NR
Gilani et al. (2014) [70]	APACHE II	0.828	χ^2^ = 5.419 (*p* = 0.712)	88.2% (DC = 19)	65.5% (DC = 19)	27.9%
Hashmi et al. (2016) [77]	APACHE II (automatic calculation using software)	0.827 (0.77–0.88)	χ^2^ = 5.46 (*p* = 0.71)	55.71%	90.21%	78.87%
Hashmi et al. (2016) [77]	APACHE II	0.823 (0.76–0.88)	χ^2^ = 11.76 (*p* = 0.16)	51.42%	90.91%	77.9%
Chang et al. (2006) [64]	APACHE II	0.82	χ^2^ = 9.8 (*p* =0.28)	NR	NR	NR
Nouira et al. (1998) [66]	APACHE II	0.82	*C* statistic χ^2^ = 25.95 (*p* < 0.001)	NR	NR	NR
			*H* statistic χ^2^ = 32.15 (*p* < 0.05)			
Liu et al. (2016) [61]	APACHE II	0.813 ± 0.055	NR	89.6% (DC ≥ 15.0)	74.8% (DC ≥ 15.0)	NR
Ratanarat et al. (2005) [68]	APACHE II	0.788	NR	80.9% (DC = 20)	63.2% (DC = 20)	NR
Khan et al. (2015) [32]	APACHE II	0.785 (0.69–0.88)	NR	94.4% (DC > 9.5)	49% (DC > 9.5)	NR
Nimgaonkar et al. (2004) [35]	APACHE II	0.77	*H* statistic χ^2^ = 123.5 (*p* < 0.05)	NR	NR	NR
Faruq et al. (2013) [37]	APACHE II	0.75 (0.67–0.82)	*C* statistic χ^2^ = 8.304 (*p* = 0.40)	NR	NR	NR
Ahluwalia et al. (1999) [29]	APACHE II	0.74	NR	93%	23.6%	NR
Chiavone and Rasslan (2005) [65]	APACHE II	0.729 (0.63–0.83)	NR	NR	NR	NR
Wilairatana et al. (1995) [82]	APACHE II	0.723	NR	77.4% (DC = 19)	61.1% (DC = 19)	70.8%
Aggarwal et al. (2006) [7]	APACHE II	0.713	*C* statistic χ^2^ = 119.3 (*p* < 0.001) *H* statistic χ^2^ = 81.1 (*p* < 0.001)	48% (39.9–56.2) (DC = 25%), 20.1% (14.1–27.3) (DC = 50%), 3.2% (1.1–7.4) (DC = 75%)	84% (79.6–88.2) (DC 25%), 96% (92.7–97.7) (DC 50%), 97.7% (98.1–100) (DC 75%)	71.9% (67.5–76) (DC 25%), 69.9% (65.1–74.3) (DC 50%), 66.8 (62.3–71.1) (DC 75%)
Halim et al. (2009) [75]	APACHE II	0.694	χ^2^ = 10.627 (*p* = 0.014)	83%	55.2%	66%
Gupta and Arora (2004) [72]	APACHE II	0.63	χ^2^ = 10.34 (*p* > 0.05)	NR	NR	89.7% (DC 70%)
Sekulic et al. (2015) [48]	APACHE II	0.623	χ^2^ = 3.05 (*p* =0.931)	Presented as a figure	81.80%	NR
Eapen et al. (1997) [30]	APACHE II (VA)	0.6068	NR	NR	NR	NR
Galal et al. (2013) [49]	APACHE II	0.6	χ^2^ = 7.34, *p* = 0.39	93% (DC = 11)	24% (DC = 11)	55.2%
Evran et al. (2016) [47]	APACHE II	Presented as a figure	NR	NR	NR	81.3%
Kiatboonsri and Charoenpan (1995) [45]	APACHE II	NR	NR	60% (DC = 50%)	95% (DC = 50%)	83% (DC = 50%)
Juneja et al. (2012) [1]	APACHE III	0.922 (0.894–0.949)	χ^2^ = 3.674 (*p* = 0.885)	78.6% (DC > 73)	86% (DC > 73)	NR
Soares et al. (2004) [40]	APACHE III	0.915 (0.898–0.933)	*H* statistic χ^2^ = 117.206 (*p* < 0.001)	NR	NR	NR
Shrestha et al. (2011) [73]	APACHE III	0.895 (0.839–0.952)	χ ^2^ = 16.904 (*p* = 0.031)	91%	73.97%	80.34%
Gilani et al. (2014) [70]	APACHE III	0.78	χ^2^ = 8.442 (*p* = 0.392)	82.3% (DC = 24)	58% (DC = 24)	NR
Wilairatana et al. (1995) [82]	APACHE III	0.694	NR	79.8% (DC = 60)	66% (DC = 60)	72.4%
Juneja et al. (2012) [1]	APACHE IV	0.928 (0.903–0.953)	χ^2^ = 8.790 (*p* = 0.360)	93.2% (cut-off point > 12.5)	66.9% (cut-off point > 12.5)	NR
Nassar et al. (2012) [42]	APACHE IV	0.883 (0.874–0.891)	*C* statistic χ^2^ = 53.7 (*p* < 0.01)	NR	NR	NR
Xing et al. (2015) [52]	APACHE IV	0.873 (0.813–0.934)	χ^2^ = 3.756 (*p* = 0.878)	NR	NR	NR
Hamza et al. (2009) [76]	APACHE IV	0.845 (0.786–0.904)	*H* statistic χ^2^ = 5.123 (*p* = 0.744)	NR	NR	NR
Evran et al. (2016) [47]	APACHE IV	Presented as a figure	NR	NR	NR	79.30%

*APACHE* Acute Physiology and Chronic Health Evaluation, *CI* confidence interval, *NR* not reported, *DC* decision criteria

**Table 6:** Model performance for all versions of SAPS

Study	Scoring system	Discrimination	Calibration	Sensitivity	Specificity	Accuracy
Ratanarat et al. (2005) [68]	SAPS I	0.746	NR	70.2% (DC = 15)	67.1% (DC = 15)	NR
Nair et al. (2016) [62]	SAPS I	0.742	NR	44.4% (DC = 61)	94.9% (DC = 61)	85.42%
Khwannimit and Bhurayanontachai (2011) [51]	SAPS II (recalibrated model)	0.919 (0.899–9.24)	*C* statistic χ^2^ = 8.6 (*p* = 0.57)	NR	NR	NR
			*H* statistic χ^2^ = 9.6 (*p* = 0.48)			
Khwannimit and Bhurayanontachai (2011) [51]	SAPS II	0.919 (0.899–9.24)	*C* statistic χ^2^ = 124.9 (*p* < 0.001)	NR	NR	NR
			*H* statistic χ^2^ = 97.5 (*p* < 0.001)			
Soares et al. (2004) [40]	SAPS II	0.916 (0.899–0.933)	*H* statistic χ^2^ = 29.400 (*p* < 0.001)	NR	NR	NR
Juneja et al. (2012) [1]	SAPS II	0.899 (0.870–0.928)	χ^2^ = 14.097 (*p* = 0.079)	83.5% (DC > 47.5)	83.5% (DC > 47.5)	NR
Godinjak et al. (2016) [31]	SAPS II	0.892 (0.84–0.94)	NR	90.2% (DC = 50.5)	75.7% (DC =5 0.5)	NR
Khawannimit and Geater (2007) [38]	SAPS II	0.888 (0.867–0.909)	*C* statistic χ^2^ = 71.44 (*p* < 0.001) *H* statistic χ^2^ = 54.01 (*p* < 0.001)	70.65% (95% CI = 65.23–75.66)	89% (95% CI = 87.08–91.02)	85% (95% CI = 82.75–86.70)
Fadaizadeh et al. (2012) [67]	SAPS II	0.887 (0.847–0.926)	*C* statistic χ^2^ = 7014 (*p* = 0:522)	83% (DC = 86.5)	77% (DC = 86.5)	NR
Sutheechet (2009) [79]	SAPS II	0.88 (0.85–0.91)	*C* statistic χ^2^ = 20.65 (*p* = 0.008)	Individual values for each risk level	Individual values for each risk level	Individual values for each risk level
Soares and Salluh (2006) [39]	SAPS II	0.88 (0.86–0.9)	*C* statistic χ^2^ = 32.136 (*p* < 0.001)	NR	NR	NR
Khan et al. (2015) [32]	SAPS II (48 hours)	0.871 (0.794–0.948)	NR	70.6% (DC > 30)	86.3% (DC > 30)	NR
Hamza et al. (2009) [76]	SAPS II	0.845 (0.787–0.903)	*H* statistic χ^2^ = 12.140 (*p* = 0.145)	NR	NR	NR
Soares et al. (2010) [41]	SAPS II	0.84 (0.81–0.87)	*C* statistic χ^2^ = 21.143 (*p* = 0.007)	NR	NR	NR
Nouira et al. (1998) [66]	SAPS II	0.84	*C* statistic χ^2^ = 73.78 (*p* < 0.001)	NR	NR	NR
			*H* statistic χ^2^ = 76.89 (*p* < 0.05)			
Sathe and Bapat (2014) [69]	SAPS II	0.83 (0.81–0.86)	χ^2^ = 26.6 (*p* = 0.001)	NR	NR	NR
Zhao et al. (2013) [50]	SAPS II	0.826 (0.803–0.85)	χ^2^ = 12.176 (*p* = 0.144)	85%	74.3%	82.4%
Zhao et al. (2013) [50]	SAPS II (48 hours)	0.821 (0.795–0.848)	χ^2^ = 11.294 (*p* = 0.186)	85%	74.3%	83.8%
Aggarwal et al. (2006) [7]	SAPS II	0.781	*C* statistic χ^2^ = 195.6 (*p* < 0.001) *H* statistic χ^2^ = 159.6 (*p* < 0.001)	46.1% (38.1–54.3) (DC = 25%), 27.35% (20.4–35.0) (DC = 50%), 10.4% (6.1–16.3) (DC = 75%)	89.3% (85.2–92.5) (DC = 25%), 95.6% (92.7–97.7) (DC = 50%), 98.7% (96.6–99.6) (DC = 75%)	74.6% (70.3–78.5) (DC = 25%), 72.3% (68.0–76.4) (DC = 50%), 68.6% (64.1–72.8) (DC = 75%)
Gilani et al. (2014) [70]	SAPS II	0.78	χ^2^ = 8.575 (*p* = 0.379)	70.5% (DC = 13)	63% (DC = 13)	NR
Zhao et al. (2013) [50]	SAPS II (initial)	0.776 (0.75–0.802)	χ^2^ = 8.332 (*p* = 0.402)	85%	74.3%	80%
Naqvi et al. (2016) [33]	SAPS II	0.75	χ^2^ = 3.724 (*p* = 0.811)	NR	NR	NR
Faruq et al. (2013) [37]	SAPS II	0.74 (0.66–0.81)	*C* statistic χ^2^ = 9.040 (*p* = 0.34)	NR	NR	NR
Khan et al. (2015) [32]	SAPS II	0.718 (0.608–0.828)	NR	70.6% (DC > 30)	60.8% (DC > 30)	NR
Wilairatana et al. (1995) [82]	SAPS II	0.71	NR	0.742 (cut-off point = 14)	0.6 (cut-off point = 14)	68.40%
Sekulic et al. (2015) [48]	SAPS II	0.69	χ^2^ = 4.41 (*p* = 0.732)	Presented as a figure	Presented as a figure	NR
Galal et al. (2013) [49]	SAPS II	0.59	χ^2^ = 7.2, *p* = 0.3	53.4% (DC = 40)	62% (DC = 40)	57.1%
Mohan et al. (2015) [46]	SAPS II	NR	NR	81.1% (DC > 35)	59.5% (DC > 35)	NR
Xing et al. (2015) [52]	SAPS 3	0.948 (0.914–0.982)	χ^2^ = 4.987 (*p* = 0.759)	NR	NR	NR
Khwannimit and Bhurayanontachai (2011) [51]	SAPS 3 (AUS) (recalibrated model)	0.917 (0.902–0.929)	*C* statistic χ^2^ = 8.2 (*p* = 0.61)	NR	NR	NR
			*H* statistic χ^2^ = 79.9 (*p* < 0.001)			
Khwannimit and Bhurayanontachai (2011) [51]	SAPS 3 (AUS)	0.917 (0.902–0.929)	*C* statistic χ^2^ = 170 (*p* < 0.001)	NR	NR	NR
			*H* statistic χ^2^ = 79.9 (*p* < 0.001)			
Khwannimit and Bhurayanontachai (2011) [51]	SAPS 3	0.914 (0.901–0.928)	*C* statistic χ^2^ = 176.3 (*p* < 0.001)	NR	NR	NR
			*H* statistic χ^2^ = 101.6 (*p* < 0.001)			
Juneja et al. (2012) [1]	SAPS 3	0.901 (0.871–0.932)	χ^2^ = 13.123 (*p* = 0.108)	76.7% (DC > 56.5)	84.7% (DC > 56.5)	NR
Soares and Salluh (2006) [39]	SAPS 3 (CSA)	0.87 (0.85–0.9)	*C* statistic χ^2^ = 9.132 (*p* = 0.33)	NR	NR	NR
Soares and Salluh (2006) [39]	SAPS 3	0.87 (0.85–0.9)	*C* statistic χ^2^ = 13.637 (*p* = 0.092)	NR	NR	NR
Silva Junior et al. (2010) [55]	SAPS 3	0.86 (0.83–0.88)	*C* statistic χ^2^ = 10.47 (*p* = 0.234)	0.75 (DC = 57)	0.86 (DC = 57)	NR
Nassar et al. (2012) [42]	SAPS 3	0.855 (0.846–0.864)	*C* statistic χ^2^ = 226.6 (*p* < 0.01)	NR	NR	NR
Soares et al. (2010) [41]	SAPS 3 (CSA)	0.84 (0.81–0.87)	*C* statistic χ^2^ = 12.608 (*p* = 0.126)	NR	NR	NR
Soares et al. (2010) [41]	SAPS 3	0.84 (0.81–0.87)	*C* statistic χ^2^ = 15.804 (*p* = 0.045)	NR	NR	NR
Hernandez et al. (2014) [78]	SAPS 3	0.8 (0.78–0.81)	*C* statistic χ^2^ = 11.5 (*p* = 0.18)	NR	NR	NR
Evran et al. (2016) [47]	SAPS 3	Presented as a figure	NR	NR	NR	81.3%
Mohan et al. (2015) [46]	SAPS 3	NR	NR	81.1% (DC > 47)	51.1% (DC > 47)	NR

*SAPS* Simplified Acute Physiology Score, *SAPS (AUS)* Simplified Acute Physiology Score (Australian), *SAPS (CSA)* Simplified Acute Physoiology Score (Central and Southern American), *CI* confidence interval, *DC* decision criteria, *NR* not reported

**Table 7:** Model performance for all versions of MPM

Study	Scoring system	Discrimination	Calibration	Sensitivity	Specificity	Accuracy
Nouira et al. (1998) [66]	MPM (24 hours)	0.88	*C* statistic χ^2^ = 29.59 (*p* < 0.001)	NR	NR	NR
			*H* statistic χ^2^ = 19.9 (*p* < 0.05)			
Nouira et al. (1998) [66]	MPM (initial)	0.85	*C* statistic χ^2^ = 36.66 (*p* < 0.001)	NR	NR	NR
			*H* statistic χ^2^ = 38 (*p* < 0.05)			
Sekulic et al. (2015) [48]	MPM II (7 days)	1.00	χ^2^ = 0.00 (*p* =1.000)	100%	Presented as figure	NR
Juneja et al. (2012) [1]	MPM II (initial)	0.928 (0.904–0.952)	χ^2^ = 8.627 (*p* = 0.375)	95.1% (DC > 27)	68.2% (DC > 27)	NR
Sutheechet (2009) [79]	MPM II (24 hours)	0.91 (0.88–0.93)	*C* statistic χ^2^ = 14.45 (*p* = 0.07)	Individual values for each risk level	Individual values for each risk level	Individual values for each risk level
Soares et al. (2004) [40]	MPM II (24 hours)	0.909 (0.891–0.926)	*H* statistic χ^2^ = 114.713 (*p* < 0.001)	NR	NR	NR
Soares et al. (2004) [40]	MPM II (initial)	0.854 (0.83–0.878)	*H* statistic χ^2^ = 373.317 (*p* < 0.001)	NR	NR	NR
Sekulic et al. (2015) [48]	MPM II (48 hours)	0.836	χ^2^ = 11.37 (*p* = 0.181)	Presented as figure	Presented as figure	NR
Sekulic et al.(2015) [48]	MPM II (72 hours)	0.817	χ^2^ = 6.04 (*p* = 0.534)	Presented as figure	Presented as figure	NR
Hamza et al. (2009) [76]	MPM II (initial)	0.81 (0.738–0.882)	*H* statistic χ^2^ = 8.825 (*p* = 0.357)	NR	NR	NR
Aggarwal et al. (2006) [7]	MPM II (24 hours)	0.733	*C* statistics χ^2^ = 73.1 (*p* < 0.001) *H* statistics χ^2^ = 69.7 (*p* < 0.001)	48.1% (39.9–56.2) (DC = 25%), 24.0% (17.5–31.6) (DC = 50%), 14.35 (9.2–20.8) (DC = 75%)	0.836 (0.789–0.876) (DC = 25%), 0.956 (0.927–0.977) (DC = 50%), 0.990 (0.971–0.998) (DC = 75%)	0.715 (0.671–0.756) (DC = 25%), 0.712 (0.668–0.754) (DC = 50%), 0.701 (0.657–0.743) (DC = 75%)
Aggarwal et al. (2006) [7]	MPM II (initial)	0.665	*C* statistics χ^2^ = 599.2 (*p* < 0.001) *H* statistics χ^2^ = 456.4 (*p* < 0.001)	27.9% (0.210–0.355) (DC = 25%), 11.4% (0.069–0.174) (DC = 50%), 2.5% (0.007–0.064) (DC = 75%)	0.920 (0.884–0.948) (DC = 25%), 0.980 (0.957–0.993) (DC = 50%), 0.993 (0.976–0.999) (DC = 75%)	0.699 (0.655–0.741) (DC = 25%), 0.682 (0.637–0.724) (DC = 50%), 0.660 (0.615–0.703) (DC = 75%)
Sekulic et al. (2015) [48]	MPM II (initial)	0.654	χ^2^ = 6.90 (*p* = 0.548)	85.20%	Presented as figure	NR
Sekulic et al. (2015) [48]	MPM II (24 hours)	Presented as figure	χ^2^ = 16.23 (*p* = 0.039)	Presented as figure	Presented as figure	NR
Juneja et al. (2012) [1]	MPM III (initial)	0.947 (0.927–0.967)	χ^2^ = 13.835 (*p* = 0.086)	94.2% (DC >19)	80.2% (DC > 19)	NR
Nassar et al. (2012) [42]	MPM III (initial)	0.84 (0.83–0.849)	*C* statistic χ^2^ = 134.2 (*p* < 0.01)	NR	NR	NR
Riviello et al. (2016) [43]	MPM III (initial)	0.72	χ^2^ = 17.66 (0.024)	NR	NR	NR
Soares et al. (2010) [41]	MPM III (24 hours)	0.71 (0.67–0.75)	*C* statistic χ^2^ = 14.242 (*p* = 0.076)	NR	NR	NR

*MPM* Mortality Probability Models, *CI* confidence interval, *NR* not reported, *DC* decision criteria

#### Discriminatory ability of models

Discrimination was reported for 104 (91.2%) of the evaluated models (Tables 5, 6 and 7). In three evaluations (two studies [45, 46]) it was reported as sensitivity and specificity only. In 101 model performance evaluations, discrimination was reported as the AUROC; in four of these evaluations AUROC was presented as a figure and a numerical value could not be ascertained [47, 48]. Where the AUROC was reported in numerical form (97 model performance evaluations) a confidence interval was only reported in 63 evaluations.

Where the AUROC was reported as a numerical value, 21 evaluations (21.7%) reported excellent discrimination. For all versions of APACHE II, SAPS II, SAPS 3 and MPM II, excellent discrimination was reported in 16.1%, 11.5%, 47.7% and 36.4% of the model evaluations respectively.

Sixty-six (68.0%) model evaluations reported very good or good discrimination; for all versions of APACHE II this was 67.7%, for SAPS II was 80.8%, for SAPS 3 was 58.3% and for MPM II it was 45.5%. Poor discrimination was reported on one occasion only, for an evaluation of SAPS II [49].

Excellent discrimination was reported more frequently when hospital mortality (*n* = 15, 25%) was the outcome in comparison to when it was ICU mortality (*n* = 6, 10%). Normal value imputation resulted in better discrimination (*n* = 4, 25% excellent and *n* = 9, 56.25% very good) than exclusion (*n* = 1, 8.33% excellent and *n* = 3, 25.0% very good) or where missing values were not reported (*n* = 16, 19.0% excellent and *n* = 32, 38.1% very good). Discrimination was better for all models with scores calculated further into the ICU stay when compared with those calculated earlier on [32, 48, 50].

Four (*n* = 2 studies) of the six evaluations with model adjustments compared them to the original model (Table 3). However an independent validation set was employed in only one study (three validations), where the models were recalibrated [51]. For all three modes (APACHE II, SAPS II and SAPS 3), recalibration resulted in the improvement of previously poor calibration; and discrimination which was already excellent remained the same.

#### Ability of models to calibrate

Only 82 (71.9%) evaluations reported calibration (Tables 5, 6 and 7). The Hosmer–Lemeshow test was reported for both *C* and *H* statistics 17 (20.7%) times, for *C* statistic only 21 (25.6%) times, for *H* statistic only nine (10.9%) times and without further detail 35 (42.7%) times.

A *p* value greater than 0.05 for the Hosmer–Lemeshow statistic was reported by 49 (59.8%) evaluations that reported calibration. For all versions of APACHE II, SAPS II, SAPS 3 and MPM II, *p* > 0.05 was reported in 60.9%, 59%, 66.7% and 50% of model performance evaluations respectively.

Ten evaluations that reported excellent discrimination also reported good calibration. Of these, three were for first-level customisations of APACHE II, SAPS II and SAPS 3 (calibration resulted in *p* < 0.05 for the Hosmer–Lemeshow statistic when the non-customised model was used) [51]. The other evaluations that reported excellent discrimination and good calibration were carried out in three studies; Juneja et al. (APACHE III, APACHE IV, MPM II (initial), MPM III (initial) and SAPS 3) [1], Sekulic et al. (MPM II at 7 days) [48] and Xing et al. (SAPS 3) [52].

A *p* value greater than 0.05 was reported more frequently when ICU mortality was the outcome (*n* = 27, 77.1%) than when hospital mortality was the outcome (*n* = 13, 27.7%). A *p* value greater than 0.05 for the Hosmer–Lemeshow statistic was obtained through exclusion of missing values 100% of the time (*n* = 3), by normal value imputation 40.9% of the time (*n* = 9) or where missing values were not reported 54.7% of the time (*n* = 29).

#### Accuracy of models

Accuracy was reported for 29 evaluations (25.0%) and ranged from 55.20 to 89.7% (Tables 5, 6 and 7).

### New model development

Three studies reported five new model developments [35, 36, 43]. These are described in Table 8. For all five new models, the AUROC was higher than that obtained with the original prognostic scoring system on which it was based. A good calibration was reported for both R-MPM and Simplified R-MPM; a poor calibration was reported for MPM-III. A poor calibration was reported for both ANN 22 and ANN 15 as well as for the original APACHE II on which they were based.

**Table 8:** New model development

Study	Ahluwalia et al. (1999) [29]	Riviello et al. (2016) [43]	Riviello et al. (2016) [43]	Nimgaonkar et al. (2004) [35]	Nimgaonkar et al. (2004) [35]
Model	New score	Rwanda MPM (R-MPM)	Simplified R-MPM	Artificial Neural Network (ANN 22)	Artificial Neural Network (ANN 15)
Source	Prospective cohort	Prospective cohort	Prospective cohort	Prospective cohort	Prospective cohort
Participants	Consecutive admissions (>13 years) to eight-bed medical ICU, India; inclusion period NR; participant age range 13–80, mean = 46	Consecutive patients (>15 years) admitted to two ICUs in different hospitals; exclusion criteria: not specified; August 2013–October 2014; participant age range 34 years (IQR 25–47) (median)	Consecutive patients (>15 years) admitted to two ICUs in different hospitals; exclusion criteria: not specified; August 2013–October 2014; participant age range 34 years (IQR 25–47) (median)	All consecutive patients (>12 years) admitted to 17-bed medical–neurological ICU, tertiary referral hospital, India; January 1996–May 1998	All consecutive patients (>12 years) admitted to 17-bed medical–neurological ICU, tertiary referral hospital, India; January 1996–May 1998
Outcomes	Hospital mortality	Hospital mortality	Hospital mortality	Hospital mortality	Hospital mortality
Predictors	1. pH (at admission); 2. serum albumin (at admission); 3. heart rate (at 48 hours); 4. GCS (at 48 hours); 5. bilirubin (at 48 hours)	Only the following five variables were included: 1. age; 2.confirmed or suspected infection within 24 hours of ICU admission; 3. hypotension or shock as a reason for ICU admission; 4. heart rate at ICU admission; 5. GSC at time of admission	Altered mental status on ICU admission (present vs not present) used in place of the GCS score in the R-MPM (see previous model)	22 APACHE II variables	15 APACHE II variables with the highest information gain (measured by calculation of entropy)
Sample size	79	427	427	2962	2962
Missing data	Not reported	Normal values attributed as in original study; two patients excluded due to lack of discharge status	Normal values attributed as in original study; two patients excluded due to lack of discharge status	Not reported	Not reported
Model development	Based on APACHE II (Knaus et al. 1985 [10]) and 11 other clinical and laboratory parameters. Backward step method used to remove non-significant (*p* > 0.05) variables (of univariate analysis)	Based on the 16 MPM III (initial) and additional variables. Variables for inclusion in model selected from the univariate analyses, based on their predictive power (as determined by *p* < 0.05) as well as their ease of capture based on experience, the proportion of missing values in the dataset, and their clinical significance	Based on the 16 MPM III (initial) and additional variables. Variables for inclusion in model selected from the univariate analyses, based on their predictive power (as determined by *p* < 0.05) as well as their ease of capture based on experience, the proportion of missing values in the dataset, and their clinical significance	Artificial Neural Network trained on an Indian patient dataset using the 22 APACHE II variables	Artificial Neural Network trained on an Indian patient dataset using the 15 APACHE II variables with the highest information gain (measured by calculation of entropy)
Model performance	Discrimination measured in terms of AUROC, sensitivity and specificity. Multivariate and univariate regression	Discrimination measured in terms of AUROC. Calibration measured as Hosmer–Lemeshow. Multivariate and univariate regression	Discrimination measured in terms of AUROC. Calibration measured as Hosmer–Lemeshow. Multivariate and univariate regression	Discrimination measured as AUROC. Calibration measured as Hosmer–Lemeshow	Discrimination measured as AUROC. Calibration measured as Hosmer–Lemeshow
Model evaluation	Developmental dataset only, no further evaluation (compared with APACHE II at 48 hours)	Internal validation with bootstrapping (compared with MPM III (initial))	Internal validation with bootstrapping (compared with MPM III (initial))	Data from 1962 patients were used to train the neural network using a back-propagation algorithm. Data from the remaining 1000 patients were used for testing this model and comparing it with APACHE II	Data from 1962 patients were used to train the neural network using a back-propagation algorithm. Data from the remaining 1000 patients were used for testing this model and comparing it with APACHE II
Results	New score ROC: 0.90, sensitivity: 98.2%, specificity: 66.6%. APACHE II (after 48 hours) ROC: 0.74, sensitivity: 92.8%, specificity: 23.6%	Rwanda MPM (R-MPM) AUROC: 0.81 (0.77–0.86), HL: χ^2^ = 11.94 (*p* = 0.154). MPM III (initial) AUROC: 0.72, HL: χ^2^ = 17.66 (*p* = 0.024)	Simplified R-MPM AUROC: 0.76, HL: χ^2^ = 11.46 (*p* = 0.177). MPM III (initial) AUROC: 0.72, HL: χ^2^ = 17.66 (*p* = 0.024)	ANN 22 AUROC: 0.87, HL *H* statistic: χ^2^ = 22.4 (*p* < 0.05). APACHE II AUROC: 0.77, HL *H* statistic: χ^2^ = 123.5 (*p* < 0.05)	ANN 15 AUROC: 0.88, HL *H* statistic: χ^2^ = 27.7 (*p* < 0.05). APACHE II AUROC: 0.77, HL *H* statistic: χ^2^ = 123.5 (*p* < 0.05)

*APACHE* Acute Physiology and Chronic Health Evaluation, *MPM* Mortality Probability Models, *ICU* intensive care unit, *GCS* Glasgow Coma Score, *IQR* interquartile range, *HL* Hosmer–Lemeshow statistic, *AUROC* area under the receiver operating characteristic

## Discussion

This systematic review of critical care prognostic models in LMICs reports good to excellent discrimination in 88.9% of evaluations between survivors and non-survivors of ICU admission and good calibration in 58.3% of those reporting calibration. In keeping with findings in HICs [3, 53], this review found good discrimination to be more frequently reported than good calibration; although good discrimination and good calibration were rarely (11.9%) reported together in the same evaluation [1, 48, 51, 52]. Three of the 10 evaluations reporting both excellent discrimination and good calibration were from recalibrated models [51], and in two [48] the sample size was small (*n* = 60). It is known that a calibration measure such as the Hosmer–Lemeshow goodness-of-fit test might demonstrate high *p* values in these circumstances, simply as a consequence of the test having lower power and not necessarily as an indication of a good fit [53].

Differences in predictors in the different models (e.g. acute diagnosis is a variable in APACEHE II but not SAPS II) and the differences in the datasets used in the various studies may have contributed to the discrepancies seen in performances of the models. Three major findings, with special relevance to the LMIC settings, limit generalisability and can affect performance: post-ICU outcomes were not available for 40.5% where ICU mortality was the outcome; only 44.8% reported a lower age limit, with 55.8% of these including patients who were aged younger than 18 years; and missing values and their handling. The original models being evaluated were developed to assess hospital mortality. Therefore, the lack of post-ICU outcome may impact on their performance, particularly as discharge from the ICU (especially in these settings) may be influenced by non-clinical discharge decisions such as shortage of ICU beds. However, post-ICU follow-up may not always be feasible in these settings due to the lack of established follow-up systems (e.g. medical registries, electronic records). Patient age may affect model performance and could be another cause for the heterogeneity seen between studies. The lower age limit for admission to adult ICUs varies between settings, perhaps resulting in the admission of paediatric patients into adult ICUs (and their subsequent use in the datasets for the validation of adult prognostic models). Twenty-three studies did not report a lower age limit for patient admission and 17 studies included patients younger than the age of 18 years; the variation in both age criteria for inclusion and for reporting make unfeasible a complete exclusion of paediatric patients from this review of adult prognostic models. Missing value handling, which can lead to bias and thus influence model performance especially in LMIC settings [53], was only reported infrequently. Where reported, imputation by normal values (which is less justifiable in LMIC settings [9]) and exclusion of incomplete records (leading to inefficient use of the dataset) were the methods frequently utilised. Research into the utility of other techniques of imputation (e.g. multiple imputation) for missing values may reduce bias and increase the interpretability of model performance. However, missing values in prognostic models in LMIC settings are likely to be a persistent problem. Some of these difficulties may be alleviated by increasing efforts to improve the availability and recording of measures such as GCS and saturations or by effecting substitutions for the measurements that are more inaccessible in LMIC settings (e.g. urea for creatinine and saturations for PaO_2_). Although two studies in this review reported the exclusion of variables [30, 50], the effect of the modifications could not be ascertained: in one case, no comparison was made with the original APACHE II model [30]; and in the second, discrimination was not reported for the simplified version of SAPS II [50]; calibration was not reported for either of these models.

Validation studies of prognostic models in LMIC settings are becoming more common; 16 of the 50 studies included were published in 2015, 2016 or 2017 and additional studies, for example Moralez et al. in Brazil [54] and Haniffa et al. [9] in Sri Lanka, have been published/awaiting publication subsequent to the literature search for this review. Consequently it is important for investigators to adhere to reporting standards, such as CHARMS—especially with regard to performance measures, outcomes and missing values— to enable better interpretation.

For a critical care prognostic model to be effective it needs to be calibrated to the target setting and have an acceptable data collection burden. However, in this review, first-level customisation was carried out in only one study [51]; the calibration of APACHE II, SAPS II and SAPS 3 models improved from poor to good and the discrimination remained excellent before and after recalibration. In HIC, medical registries enable standardised, centralised, often automated, electronic data gathering, which can then be validated; thus reducing the burden of data collection. These registries include mechanisms for providing feedback on critical care unit performance and also enable regular recalibration of prognostic models, thus minimising the incorrect estimation of predicted mortalities due to changes in case mix and treatment. The absence of such registries in LMIC settings, with important exceptions (e.g. in Brazil, Malaysia and Sri Lanka), is a significant barrier for the validation and recalibration of existing models, and the development of models tailored to these settings. Accordingly, none of the validation studies included in this review is an output from a medical registry, no studies reported on model performance from different time points in the same setting and only three studies were conducted in two or more hospitals [41, 43, 55].

The use of prognostic models in practice is thought to be influenced by the complexity of the model, the format of the model, the ease of use and the perceived relevance of the model to the user [56]. The development of models with fewer and more commonly available measures perhaps in conjunction with medical registries promoting research may also be effective in improving mortality prediction in these settings; for example, the simplified Rwanda MPM [43] and TropICS [57]. Introducing simple prognostic models like those already mentioned and emphasising their usefulness by providing output that is relevant to clinicians, administrators and patients is therefore more likely to result in the collection of required data and their application in a clinical context.

ICU risk prediction models need to exhibit good calibration before they can be used for quality improvement initiatives [58, 59]. Setting-relevant models such as TropICS [57], which are well calibrated, can be used for stratification of critically ill patients according to severity, which is a pre-requisite for impact assessment of training and other quality improvement initiatives. However, models that show poor calibration but have a good discriminatory ability may still be of benefit if their intended use is for identifying high-risk patients for diagnostic testing or therapy and/or for inclusion criteria or covariate adjustment in a randomised controlled trial [58, 59].

### Limitations

This review was limited to a single database (PubMed). There is no MeSH for LMIC (non-HIC) and hence a hand search strategy was deployed. No attempt was made to distinguish between upper and lower middle-income countries which are very heterogeneous in terms of provision, resources and access to healthcare. The review was intended to be for adult prognostic models used only in adult patients; however, due to the manner in which the studies were reported it was not possible to exclude paediatric patients.

## Conclusion

Performance of mortality risk prediction models for ICU patients in LMICs is at most moderate, especially with limitations in calibration. This necessitates continued efforts to develop and validate LMIC models with readily available prognostic variables, perhaps aided by medical registries. Robust interpretations of their applicability are currently hampered by poor adherence to reporting guidelines, especially when reporting missing value handling.

## Additional files

Additional file 1: A table presenting the search terms used. (XLSX 27 kb)

Additional file 2: A table presenting the checklist for critical appraisal and data extraction for systematic reviews of prediction modelling studies. (XLSX 41 kb)
